# The Causal Effect of Reproductive Factors on Breast Cancer: A Two-Sample Mendelian Randomization Study

**DOI:** 10.3390/jcm12010347

**Published:** 2023-01-01

**Authors:** Lijun Jia, Wei Lv, Liang Liang, Yuguang Ma, Xingcong Ma, Shuqun Zhang, Yonglin Zhao

**Affiliations:** Department of Oncology, The Second Affiliated Hospital of Xi’an Jiaotong University, No. 157 Xiwu Road, Xi’an 710004, China

**Keywords:** breast cancer, reproductive factors, mendelian randomization, GWAS, causal effect

## Abstract

Several studies have shown that female reproductive factors are associated with breast cancer (BC), but the results differ. We conducted two-sample MR in the present work. The raw data applied in the MR study were all from the Genome-wide association study (GWAS) database. The causal effect of reproductive factors on breast cancer were mainly estimated by the standard inverse variance weighted (IVW) method. Cochran’s Q test and I^2^ statistics were used to assess heterogeneity. The pleiotropy was evaluated by MR-Egger intercept test and MR-PRESSO. Finally, the leave-one-out analysis was performed to evaluate the robustness of the MR results. We found that there was a negative causal effect of the age at last live birth on BC (OR = 0.687, 95%CI = 0.539–0.875, *p* = 0.002) and positive effect of the age at menopause on BC (OR = 1.054, 95%CI = 1.034–1.075, *p* = 8.010 × 10^−8^). Additionally, there were null effects of the age at menarche (OR = 0.977, 95%CI = 0.915–1.043, *p* = 0.484), the age at first sexual intercourse (OR = 1.053, 95%CI = 0.958–1.157, *p* = 0.284) and the age at first birth (OR = 0.981, 95%CI = 0.936–1.027, *p* = 0.404) on BC. All these results were reliable and stable. In conclusion, the present study showed that younger age at last birth and older age at menopause could increase the risk of BC.

## 1. Introduction

Breast cancer (BC) is a common tumor in women, and it is also the main cause of death in women caused by cancer according to Global Cancer Statistics 2020; it was estimated there were about 2.3 million incident cases and 685,000 deaths [1,2]. In the last decades, there is increasing incidence of breast cancer among most countries due to increased life expectancy and lifestyle changes [3,4,5]. Consequently, it is of great concern for researchers to figure out any risk factors for BC prevention.

Female reproductive factors are heritable traits that vary widely between individuals. They are associated with many chronic diseases and cancers [6]. Previous studies have been widely investigated with regards to the association between reproductive factors and many diseases such as lung cancer, melanoma, hepatocellular carcinoma, Parkinson’s disease and cardiovascular disease [7,8,9,10,11]. In recent years, the role of reproductive factors in the incidence of BC have caught more and more attention, and there were many studies focused on the association between reproductive factors and breast cancer, with different and conflicting results [12,13,14,15,16]. Moreover, many of these studies were case-control or observational studies, in which many confounders and biases could not be eliminated; the reverse causality could not be avoided, either. Thus, the causal relationship between reproductive factors and BC remains controversial and unclear. It is necessary to elucidate this causality for BC prevention. Prospective studies and randomized controlled trials are instructive for causality assessment, but they are time-consuming, laborious, and sometimes, unethical.

Mendelian randomization is a novel epidemiological method for causality investigation, which has gained increasing attention in the last decades and widely used in many studies [17]. To our best knowledge, there are no studies that explored the causal relationship between reproductive factors and BC. Therefore, in this study, we are the first to used MR analysis, which uses genetic variants to identify the causality between female reproductive factors and the risk of BC.

## 2. Materials and Methods

### 2.1. Study Design

**Figure 1:**
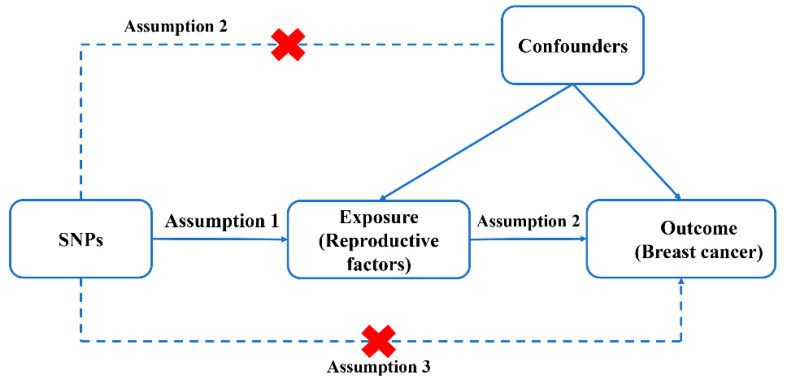
Diagram for Mendelian randomization (MR). MR is based on three hypotheses. First, SNPs selected as IVs should be closely related with exposure; second, selected SNPs must be independent of confounders; third, IVs are associated with BC (outcome) only via reproductive factors (exposure) rather than through a direct association.

A two-sample MR was conducted in the present study by Single nucleotide polymorphisms (SNPs) obtained from GWAS summary data. Reproductive factors were selected as exposure, while BC was selected as outcome. There are three assumptions that should be met for SNPs selected as instrumental variables (IVs) in MR analysis [18]: firstly, SNPs selected as IVs must be closely related to exposures; secondly, IVs must be free of confounders; finally, IVs have an impact of outcome only via exposure rather than through a direct effect (Figure 1).

### 2.2. Data Sources

Five reproductive factors demonstrated by previous studies were involved in the present study. The IV with regard to the age at menarche was extracted from a GWAS study, which consisted of 182,416 women of European descent from 57 studies and 2,441,816 single nucleotide polymorphisms (SNPs) were included [19]. IVs on age at first sexual intercourse (AFS) and age at first birth (AFB) were extracted from a GWAS study conducted by Mills MC [20], in which 16,359,424 and 9,702,772 SNPs were identified, respectively. The data of age at last live birth (ALB) were obtained from recent, publicly available GWAS data published by Ben Elsworth involving 170,248 individuals of European descent “https://gwas.mrcieu.ac.uk/datasets/” (accessed on 1 November 2022). The GWAS summary data for age at menopause were derived from a large-scale genomic analysis including 69,360 women of European ancestry and 2,418,696 associated SNPs were identified [21]. The summary-level data related to breast cancer were obtained from a GWAS study consisting of 76,192 cases and 63,082 controls of European ancestry [22].

### 2.3. IVs Extraction

Firstly, significant SNPs (*p* < 5 × 10^−8^) were extracted as the potential instrumental variables (IVs). Then, to avoid biases caused by linkage disequilibrium (LD) [23], a linkage disequilibrium correlation coefficient r^2^ (r^2^ < 0.001), and a number of bases between two SNPs (kb > 5000) were set. The MR-PRESSO was performed to identify any potential outliers. The Steiger filtering test was also performed to avoid the reverse causality. Finally, weak IVs with an *F*-statistic < 10 were excluded and then the remaining SNPs were selected as IVs for further MR analysis.

### 2.4. Statistical Analysis

The causal effect of reproductive factors on BC were mainly estimated by the standard inverse variance weighted (IVW) method [24], while the MR-Egger, weighted median, simple mode and weight mode methods were also performed as supplementary analysis. Cochran’s Q test and I^2^ statistics were used to assess heterogeneity [25]. The pleiotropy was evaluated by the MR-Egger intercept test. Finally, the robustness of MR analysis results were assessed by the leave-one-out test [26]. All data analyses were conducted in TwoSampleMR packages in R version 4.1.2. The differences were considered to be statistically significant when *P*-value < 0.05.

## 3. Results

### 3.1. Genetics Variants Selection

After removing the SNPs which were palindromic with intermediate allele frequencies, weak IVs and IVs that explain more of the variance in the outcome than in the exposure, there were 64,173,57,41,6 SNPs with regards to age at menarche, AFS, AFB, age at last live birth and age at menopause that were extracted for further MR. The *F*-statistic of these SNPs are all greater than 10, fulfilling the assumption of strong relevance for MR studies. Detailed information about all SNPs is shown in Supplementary Files S1–S5.

### 3.2. Causal Effect of Age at Menarche and Menopause on BC

**Figure 2:**
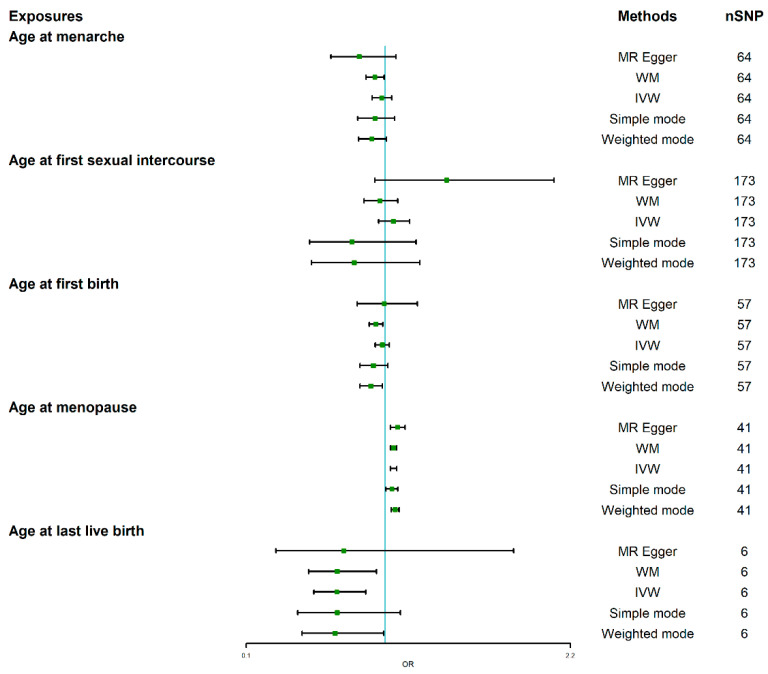
Forrest plot of the causal effects of five reproductive factors on BC. BC: breast cancer.

We firstly assessed the causal effect of age at menarche on BC. The results are summarized in Table 1 and Figure 2. The causal association evaluated by IVW showed that there was null causal effect of age at menarche on BC (OR = 0.977, 95%CI: 0.915 to 1.043; *p* = 0.484). The MR Egger (OR = 0.832, 95%CI: 0.648 to 1.069; *p* = 0.156), simple mode (OR = 0.934, 95%CI: 0.822 to 1.061; *p* = 0.298) and weighted mode (OR = 0.914, 95%CI: 0.829 to 1.008; *p* = 0.076) also verified this result, indicating that the age at menarche has no causal association with BC.

**Figure 3:**
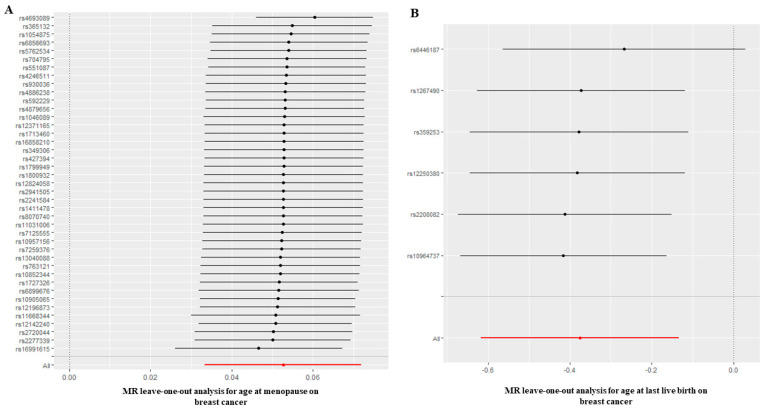
(**A**) Leave-one-out analysis plots for age at menopause on BC. (**B**) Leave-one-out analysis plots for age at last live birth on BC. BC: breast cancer.

Furthermore, we assessed the causal effect of age at menopause on BC. Interestingly, the causal relationship assessed by IVW suggested there was a negative causal effect of age at menopause on BC (OR = 1.054, 95%CI: 1.034 to 1.075; *p* = 8.010 × 10^−8^) (Table 1 and Figure 2), and this causality was further verified by MR Egger (OR = 1.080, 95%CI: 1.034 to 1.129; *p* = 1.404 × 10^−3^), weighted median (OR = 1.054, 95%CI: 1.034 to 1.074; *p* = 7.270 × 10^−8^), simple mode (OR = 1.043, 95%CI:1.005 to 1.082; *p* = 3.359 × 10^−2^) and weighted mode (OR = 1.064, 95%CI: 1.039 to 1.090; *p* = 1.050 × 10^−5^) methods. The heterogeneity tested by Cochran’s Q test and I^2^ statistics showed that heterogeneity existed (Q = 122.72; *p* = 2.468 × 10^−8^; I^2^ = 67.406), but no pleiotropy (MR-Egger intercept = −5.324 × 10^−3^; SE = 0.00438; *p* = 0.232), and the leave-one-out test showed that the results were reliable and stable (Figure 3A). Based on a previous study [27], when there was heterogeneity presence but pleiotropy absence, weighted median methods were recommended to be applied, which indicates that the older the age at menopause, the higher the risk of BC.

### 3.3. Causal Effect of AFS on BC

We next evaluated the causal association between AFS and BC. The results are also summarized in Table 1 and Figure 2. There was null causal association between AFS and BC analyzed by IVW (OR = 1.053, 95%CI: 0.958 to 1.157; *p* = 0.284), and this is confirmed by other MR analysis methods, including MR Egger (OR = 1.398, 95%CI: 0.934 to 2.093; *p* = 0.105), weighted median (OR = 0.966, 95%CI: 0.862 to 1.082; *p* = 0.546), simple mode (OR = 0.784, 95%CI: 0.512 to 1.201; *p* = 0.265) and weighted mode (OR = 0.800, 95%CI: 0.523 to 1.224; *p* = 0.305), which also indicates that there was null causal effect of AFS on BC.

### 3.4. Causal Effect of Age at First Birth and Last Live Birth on BC

Finally, we assessed the causal association of age at birth on BC. The causal relationship assessed by IVW showed there was null causal effect of AFB on BC (OR = 0.981, 95%CI: 0.936 to 1.027; *p* = 0.404), and similar results were analyzed by other MR methods including MR Egger (OR = 0.994, 95%CI: 0.818 to 1.209; *p* = 0.956) and simple mode (OR = 0.923, 95%CI: 0.837 to 1.017; *p* = 0.111) (Figure 2). Interestingly, the causal association assessed by IVW showed that with 1SD decrease in AFB, the risk of BC could reduce by about 31.3% (OR = 0.687, 95%CI: 0.539 to 0.875; *p* = 0.002); weighted median also verified the results (OR = 0.690, 95%CI: 0.504 to 0.944; *p* = 0.020) (Table 1, Figure 2), indicating that the younger the age at first birth, the lower the risk of BC. The Cochran’s Q test showed that there was no heterogeneity (Q = 2.553; *p* = 0.768), and the MR-Egger intercept (MR-Egger intercept = −1.564 × 10^−3^; SE = 0.0112; *p* = 0.896) and MR-PRESSO [27] both showed that there was no pleiotropy, and the results were reliable and stable revealed by the leave-one-out test (Figure 3B).

## 4. Discussion

Multiple studies have investigated the association between female reproductive factors and BC, but discrepancies existed in the reported results. Some findings suggest that early menarche increases the risk of breast cancer [13,14,16,28]. However, other studies have different results. Khincha [12] drew a conclusion that age at menarche and oral contraceptive use do not affect breast cancer risk in a retrospective observational study consisting of questionnaire data from 152 women. Arthur also found no association between early menarche and breast cancer risk in a case-controlled study [29]. Our findings are consistent with Arthur’s, indicating that there was no causal relationship between age at menarche and BC.

Numerous studies demonstrated that the older the age at first birth, the higher the risk of BC, particularly for ER-positive tumors [30,31,32]. However, we did not observe this association in our study. The inconsistency of the results may be due to the fact that BC data we included did not stratify according to hormone receptor or histology. The association between age at first birth and BC risk was weakened by other types of tumors. Another view, that the duration of the interval from menarche to first birth, rather than isolated age at menarche or age at first birth impacts BC risk, would also explain the difference [33,34]. The undifferentiated breast tissue is susceptible to carcinogens in the duration from menarche to first birth.

It is well established that younger age at first sexual intercourse is a risk factor for cervical cancer [35,36]. However, the relationship between age at first sexual intercourse and BC was rarely studied. The present work does not suggest a causal association between age at first sexual intercourse and BC.

ALB and the risk of BC are not fully elucidated. Some studies found a positive association between ALB and BC [37,38]. Others found no association between ALB and BC [39,40]. However, our Mendelian randomization study results suggest ALB was negatively associated with the risk of BC. This is a result worthy of further investigation and discussion.

Menopause marks the end of a woman’s reproductive life span and the cessation of endogenous hormone production. Previous studies have consistently suggested that late age at menopause is a risk factor for BC [41,42,43,44]. Our study similarly confirmed a positive causal association between age at menopause and BC risk. According to our study, women who are younger at last birth and older at menopause are recommended to perform breast cancer screening tests earlier on account of a higher risk of BC.

Compared to previous studies, our study included several notable advantages. First, the study broadens the scope of the existing literature by including multiple large-scale GWAS summary statistics. Moreover, a key strength of this study was that the MR analysis method we conducted is not vulnerable to confounding factors, ensuring the reliability of the causal association between reproductive factors and BC risk.

However, we acknowledge some limitations to our study. First, the data we included were derived from questionnaires and have recall bias. Second, our data was not stratified by age and BC type, so the effect of reproductive factors on different types of BC risk in women of different ages could not be obtained. Third, the genetic instruments comprising SNPs significantly associated with age at first sexual intercourse consisted of both genders; however, the outcome GWAS data of BC were made up of female data only. The MR analyses could have been improved if gender composition of exposure and outcome are the same.

## 5. Conclusions

According to our study, young age at last birth and older age at menopause could increase the risk of BC, while age at menarche, age at first sexual intercourse and age at first birth are not causally associated to breast cancer. These conclusions can provide a reference for family planning.

## Figures and Tables

**Table 1:** Association of Reproductive factors with BC using various methods.

Exposure	Method	*p*-Value	OR	95%LCI	95%UCI
Age at menarche	MR Egger	0.156	0.832	0.648	1.069
	WM	0.030	0.933	0.876	0.993
	IVW	0.484	0.977	0.915	1.043
	Simple mode	0.298	0.934	0.822	1.061
	Weighted mode	0.076	0.914	0.829	1.008
Age at first sexual intercourse	MR Egger	0.105	1.398	0.934	2.093
	WM	0.546	0.966	0.862	1.082
	IVW	0.284	1.053	0.958	1.157
	Simple mode	0.265	0.784	0.512	1.201
	Weighted mode	0.305	0.800	0.523	1.224
Age at first birth	MR Egger	0.956	0.994	0.818	1.209
	WM	0.009	0.939	0.896	0.984
	IVW	0.404	0.981	0.936	1.027
	Simple mode	0.111	0.923	0.837	1.017
	Weighted mode	0.019	0.906	0.837	0.982
Age at last live birth	MR Egger	0.541	0.731	0.292	1.832
	WM	0.020	0.690	0.504	0.944
	IVW	0.002	0.687	0.539	0.875
	Simple mode	0.179	0.69	0.434	1.098
	Weighted mode	0.100	0.676	0.462	0.990
Age at menopause	MR Egger	1.404 × 10^−3^	1.080	1.034	1.129
	WM	7.270 × 10^−8^	1.054	1.034	1.074
	IVW	8.010 × 10^−8^	1.054	1.034	1.075
	Simple mode	3.359 × 10^−2^	1.043	1.005	1.082
	Weighted mode	1.050 × 10^−5^	1.064	1.039	1.090

WM: weighted median; IVW: inverse-variance weighted; OR: odds ratio; LCI: lower confidence interval; UCI: upper confidence interval; BC: breast cancer.

## Data Availability

The original contributions presented in the study are included in the article/supplementary material, and further inquiries can be directed to the corresponding author.

## Supplementary Materials

The following supporting information can be downloaded at: https://www.mdpi.com/article/10.3390/jcm12010347/s1, The Supplementary Material for this article contains the calculation processes of Mendelian randomization and detailed information of SNPs.
